# BLISS: biding site level identification of shared signal-modules in DNA regulatory sequences

**DOI:** 10.1186/1471-2105-7-287

**Published:** 2006-06-07

**Authors:** Hailong Meng, Arunava Banerjee, Lei Zhou

**Affiliations:** 1 Department of Computer and Information Science and Engineering, College of Engineering, University of Florida, Gainesville, FL 32611, USA; 2 Department of Molecular Genetics and Microbiology, UF Shands Cancer Center, College of Medicine, University of Florida, Gainesville, FL 32610, USA

## Abstract

**Background:**

Regulatory modules are segments of the DNA that control particular aspects of gene expression. Their identification is therefore of great importance to the field of molecular genetics. Each module is composed of a distinct set of binding sites for specific transcription factors. Since experimental identification of regulatory modules is an arduous process, accurate computational techniques that supplement this process can be very beneficial. Functional modules are under selective pressure to be evolutionarily conserved. Most current approaches therefore attempt to detect conserved regulatory modules through similarity comparisons at the DNA sequence level. However, some regulatory modules, despite the conservation of their responsible binding sites, are embedded in sequences that have little overall similarity.

**Results:**

In this study, we present a novel approach that detects conserved regulatory modules via comparisons at the binding site level. The technique compares the binding site profiles of orthologs and identifies those segments that have similar (not necessarily identical) profiles. The similarity measure is based on the inner product of transformed profiles, which takes into consideration the p values of binding sites as well as the potential shift of binding site positions. We tested this approach on simulated sequence pairs as well as real world examples. In both cases our technique was able to identify regulatory modules which could not to be identified using sequence-similarity based approaches such as rVista 2.0 and Blast.

**Conclusion:**

The results of our experiments demonstrate that, for sequences with little overall similarity at the DNA sequence level, it is still possible to identify conserved regulatory modules based solely on binding site profiles.

## Background

Transcription is the fundamental biological process in which a selected region of DNA is transcribed into RNA by a molecular machinery, a core component of which is RNA polymerase. For most protein-coding genes, transcription is the intermediate step via which the information coded in their DNA is "expressed" into functioning proteins. For others, such as RNA genes, the product of the transcription itself may have biological function. Even though each cell has the complete set of genes in its chromosomal DNA, only a portion of the genes are transcribed (expressed) in any particular cell depending on tissue/cell type, developmental stage, etc [1]. The transcriptome, that is all of the genes that are selectively transcribed in a cell, determines the function and morphology of the cell. In addition, the level (i.e., rate) of transcription is often regulated in response to intra-cellular and extra-cellular environmental factors to achieve cellular homeostasis. Normal transcriptional regulation, i.e., the right genes being transcribed at the right times, in the right cell, and at the appropriate rates, is central to almost all physiological processes. Abnormal regulation of transcription often results in disruption of development and/or pathological states. For example, ectopic (i.e., abnormally high) expression of oncogenes leads to hyperplasia and cancer.

A basic element of transcriptional regulation is the interaction of transcription factors (*trans *factors) and their corresponding transcription factor binding sites (TFBSs, also referred to as *cis *factors) on the DNA. Transcriptional regulation of a gene (e.g. restricted transcription in a particular cell type, or elevated transcription, in response to UV light) is often mediated through the functional/physical interactions among multiple transcription factors, each recruited to the proximity of the DNA in part by their selective affinity to their corresponding binding sites. For example, the *even-skipped*(*eve*) gene is transiently expressed in 7 alternative stripes on the longitudinal axis in the developing *Drosophila melanogaster *embryo at the blastoderm stage. Each of the seven stripes is regulated by a distinct set of transcription factors binding to their corresponding binding sites located in a DNA segment flanking the even-skipped gene. The most well investigated of these segments is the stripe 2 regulatory region, which has identified binding sites for 5 different transcription factors in a 700 bp (base pair)-1 kb (kilo base pair) DNA region in front of the transcription initiation site of the *eve *gene. Evolutionary comparison of this transcription regulatory module in different *Drosophila *species has revealed that most of the binding sites are highly conserved and functional, even though the underlying DNA sequence has undergone considerable change [2].

A useful analogy to understanding the composition of DNA regulatory modules is to consider DNA as a sequence of "Letters" and individual binding sites as "Words". Then, a functional module of closely associated binding sites can be conceived of as the "Sentence" command embedded in the DNA sequence that guides transcription. The problems associated with identifying the "Sentence" commands in a DNA sequence are two fold. First, the binding sites are degenerate in nature, that is, the same "Word" may have different letters in certain positions. Second, the "Words" are themselves interspersed by varying lengths of meaningless "Letters".

One approach to identifying DNA regulatory modules is through cross-genome comparison. The assumption underlying this approach is that DNA sequences encoding functional TFBSs are under selective pressure to be conserved during evolution, whereas non-functional DNA mutate/change more rapidly. Thus, if DNA sequences flanking orthologs in two related species were to be compared for sequence-level similarity, DNA regulatory modules would appear as conserved "islands" in a sea of otherwise not-conserved DNA sequences. Approaches in this category include rVista2.0, ConSite, PhyME, TOUCAN, CREME, TraFAC, etc [3-10]. For instances, based on the sequence level conservation between human and mouse, Cora et al. predicted functional TFBSs that are statistically over-represented and share the same specific Gene Ontology (GO) terms [9]. This kind of cross-genome comparison approaches has successfully led to the discovery of regulatory modules that were subsequently verified by functional characterization [11].

The disadvantage of the sequence-based approach is that it is dependent on the overall conservation of the DNA region harbouring the regulatory module. As we mentioned earlier, TFBS sequences are degenerate in nature and are interspersed by non-functional sequences which are not under conservation pressure. Depending on the ratio of functional versus non-functional base-pairs in the DNA region, it is entirely possible that the overall sequence level conservation of the region be indistinguishable from the background level, while the actual TFBSs making up the functional module still be conserved. In other words, it is possible that despite the conservation of the "Sentence" command at the **binding site **level, the overall conservation of the DNA backbone at the **sequence **level be minimal or non-detectable. This situation is aggravated if the participating binding sites are highly degenerate (i.e., tolerate many variations at the sequence level) and the spaces between the binding sites are long. In fact, it has been observed by researchers in many instances that while the regulatory region has no detectable overall similarity, the transcription regulatory function is preserved [12]. Sequence-based approaches, or approaches requiring filtration of sequences based on DNA level similarity, are not helpful for identifying the responsible TFBSs in this scenario.

Since the conservation pressure is at the binding site level, i.e., the sequence must be able to maintain binding affinity to the transcription factor(s), it makes biological sense to perform comparisons at the binding site level rather than at the sequence level. This, however, is currently hindered by two factors. The first limitation is the effectiveness of identifying transcriptional binding sites in a given DNA sequence. The set of TFBSs for a given TF can be quantitatively represented using a frequency matrix that describes the binding specificity of the TF at each of its positions. The quality of the matrix used to identify potential TFBSs is determined by the number and quality of known binding site sequences used to construct the matrix. As a result of the development of new technologies such as Chip [13] and ChIP-chip [14], it is anticipated that binding site instances will be identified at a unprecedented rate which will undoubtedly greatly enhance both the quality as well as the coverage of binding site matrices in the near future [15].

The second limitation is that we currently do not understand the grammar governing how binding sites (Words) make up the regulatory modules (Sentences). Based on our understanding of transcriptional regulation, such a grammar should have at least three components: (1) the composition of the binding sites, (2) the sequence of the binding sites, and (3) the spaces between/among the binding sites. Currently, the number of regulatory modules that have been thoroughly characterized is far fewer than what is required to decode this grammar.

A major obstacle for biologists working on transcriptional regulation is to locate and identify potential TFBSs responsible for a particular regulatory module, especially in sequences that do not have significant conserved islands. In this paper, we describe a novel methodology for binding site level identification of conserved regulatory modules in such sequences.

## Results

### Simulating sequence pairs harbouring a conserved module of binding sites

Since the number of well-studied regulatory modules is currently rather sparse, we chose to simulate sequence sets (pairs) representing the domain of our interest, i.e., conserved binding site patterns in a pair of sequences which nonetheless have little or no similarity at the sequence level. In many cases, experimental investigation in a model organism has narrowed down the location of the regulatory module(s) for a particular gene to a relatively short region (e.g. within 1 kb), whereas for the ortholog in a less-studied organism (reference organism), information about the localization of the module is absent (except that it is generally in the proximity of the gene). In view of this, in the first (current) stage of the development of BLISS, we considered the identification of a conserved module present in both a short sequence of about 100–500 bps, representing the model organism, and a longer sequence (5–6 kb), representing the reference organism. Although this simplification limits the applicability of the current methodology, it does highlight the promise of our approach.

For each sequence pair, the backbones for both the short sequence and the long sequence were generated randomly and thus had no sequence similarity. A hypothetical module involving binding sites for 4–8 distinct transcription factors was first introduced into the short sequence. The binding site sequences were randomly selected from the instances recorded in the TRANSFAC 9.1 database [15]. The rules governing the formation of the hypothetical module were as follows:

1.) A module contains binding sites for 4–8 distinct transcription factors.

2.) For each transcription factor, there may be more than one binding site in the module.

**Figure 1:**
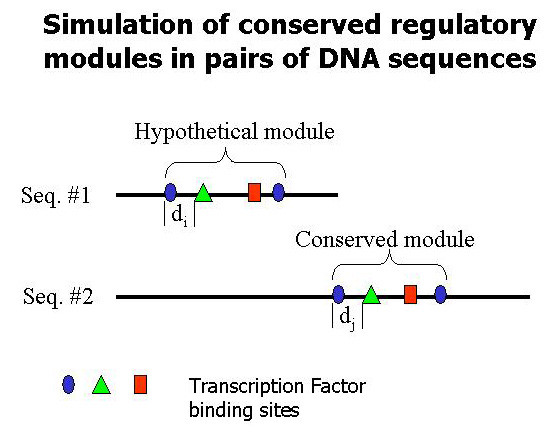
**Simulating conserved regulatory modules in a pair of random sequences**. Each pair has a short (100–500 bp) and a long (5–6 kb) sequence. For both sequences the backbones were generated randomly. The hypothetical module was formulated according to rules described in the text and inserted into the short sequence. A "conserved" module with binding sites corresponding to the same transcription factor, but with different sequences was inserted into the long sequence. The distances between consecutive binding sites were also different between the hypothetical module and the conserved module.

3.) The distance between consecutive binding sites is "*d*_*i*_", where in 65% of the cases *d*_*i *_lies within 5–20 bps, in 22% of the cases *d*_*i *_lies within 21–50 bps and in the remaining 13% of the cases *d*_*i *_lies within 51–60 bps (Figure 1).

The range of values for *d*_*i *_was based on background knowledge as well as a statistical analysis of the distances between pairs of TFBSs in TransCompel database by Qiu et al. which revealed the above distribution of distances between pairs of TFBSs [16].

The hypothetical module was first simulated according to the above rules in the short sequence. Subsequently, a "conserved" module was formulated and inserted into the longer sequence at a random location. The rules governing the formation of the "conserved" module were as follows:

1.) It is comprised of TFBSs that correspond to the same transcription factors as present in the hypothetical module.

2.) The sequence for each TFBS is randomly chosen from the recorded instances in TRANSFAC 9.1, with the caveat that it cannot be the same instance(s) that was (were) used to construct the hypothetical module in the short sequence.

3.) The respective order of TFBSs is the same as in the hypothetical module in the short sequence.

4.) The distance between consecutive binding sites in the conserved module is *d*_*j*_; *d*_*j *_is a function of *d*_*i *_in that *d*_*j *_lies in the range (*d*_*i *_- Δ*d*, *d*_*i *_+ Δ*d*) (Figure 1).

Δ*d *is the "perturbation factor" – the variation of distance between corresponding binding sites in the hypothetical module and the conserved module. In this study, we used Δ*d *= 4 (See Discussion). A total of 10,000 pairs of sequences were generated according to above rules, and were used to test and evaluate various algorithms.

### Identifying a conserved module by comparison at the binding site level

As stated above, the objective of our methodology is to identify conserved regulatory modules within highly divergent sequences. The sequence pairs in our simulated data-set had little overall sequence similarity. Of the 10,000 pairs, 73.32% have no similarity detectable by BLAST analysis (E = 0.01, Blast2seq). This indicated that the conservation of binding sites in the hypothetical module and the conserved module was not sufficient to allow detection at the sequence level. Of those that did have a significant match, the output alignments were shorter than 30 bps, which was far shorter than the length of the inserted module.

#### M_score

**Figure 2:**
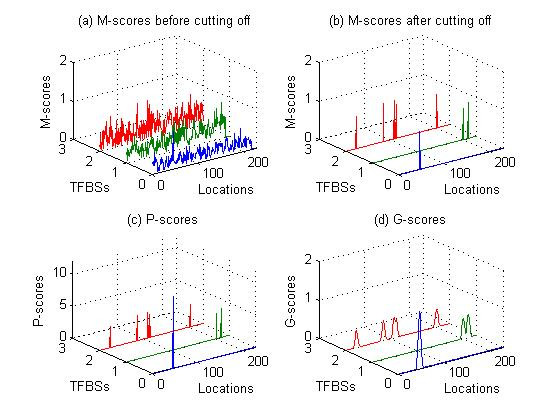
**Gaussian smoothing of the TFBS profile**. a.) Profile of matrix matching scores (*M*_*score*) for three TFBSs along a short DNA sequence. b.) *M*_*score *profile after applying a cut_off of 0.75. c.) Profile of *P_score *after incorporating the p value of binding site matches. d.) Profile of the *G*_*score *after Gaussian smoothing. The colors represent three different TFBSs: Red, En; Green, Croc; Blue, Lun-1.

To identify the conserved module at the binding site level, we first generated the potential TFBS profiles for each of the simulated sequence pairs. A matrix scoring method similar to that used in Match [17] was implemented (see Methods), which allowed us to score each sequence against the frequency TFBS matrices recorded in TRANSFAC 9.1 (*M*_*score*, Figure 2a). When a cut-off value of 0.75 (on *M*_*score*) was applied, on average there were about 3000 identified potential TFBSs in every thousand base pairs of simulated sequences. This is similar to what we observed when using genomic DNA sequences randomly extracted from model organism databases (data not shown).

To identify the hypothetical module embedded in the sequences, we tried several different algorithms that compared the binding site profiles of the short and the long sequences. Of those tested, a scoring method (using inner-products) based on a statistical evaluation of binding site matches after a Gaussian smoothing of the binding site profiles gave reliable and promising results.

#### P_score

The matrix score (*M*_*score*), by virtue of its definition (f.1), ranges from 0 to 1 for all TFBS matrices. Thus it does not differentiate short and relatively simple matrices that match DNA sequences with a high frequency from those long and stringent matrices that match DNA sequences only rarely. For example, the binding site for En (I$ED_06) has 7 positions, and on average there are 320 matches (with *M*_*score *> 0.75) on any 10,000 bp random sequence. In contrast, the binding site for Bel-1 (V$BEL1_B) has 13 positions, and the average number of matches with *M*_*score *> 0.75 in a 10,000 bp random sequence is 3. It is clear that a match involving the binding site V$BEL1_B is far more significant than a match with I$ED_06. To differentiate this, we introduced the p value of the *M*_*score*, which was estimated by calculating the fraction of randomly generated sequences that have scores equal to or higher than that *M*_*score*. We then calculated the *P_score *(see Methods) as the product of -log (p value of *M*_*score *> cut_off) and the *M*_*score *(Figure 2b).

#### G_score

To account for the variation in the distances between/among binding sites, we performed a Gaussian smoothing of the *P_score *(see Methods). Through empirical testing (data not shown), we found that a variance of σ^2 ^= 9 gave the best performance. We denote the Gaussian smoothed score profile as the *G*_*score *profile of the sequence (Figure 2d).

#### BLISS_score

*G*_*score *profiles were generated for both the short and the long sequences. To identify a maximum match at the binding site profile level, the short *G*_*score *profile was slid along the long *G*_*score *profile. At each position, the match between the short profile and its corresponding region of equal length (length of the window) in the long profile was evaluated using an inner-product as the *BLISS*_*score *(see Methods).

**Figure 3:**
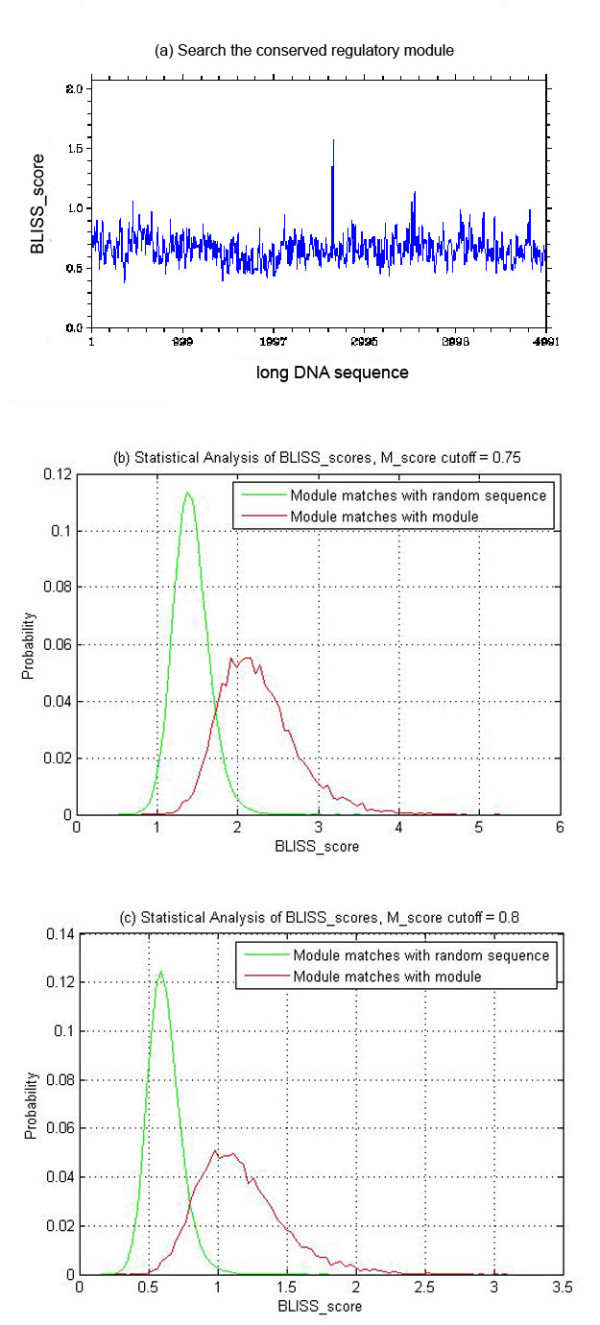
**Identifying a conserved module at the binding site level**. a.) The *G*_*score *profile is slid along the long sequence to calculate the *BLISS*_*score*. The peak in the *BLISS_Score *profile represents the maximum match at the binding site level, and in this case, contains the match of the conserved hypothetical regulatory module simulated in the sequences. b.) and c.) show the distribution of *BLISS*_*score *in true matches vs. background with a cut_off value of 0.75 and 0.80, respectively.

Note that since the "length of the window" appears in the denominator, the *BLISS*_*score *is independent of the length of the short profile (or the length of the window). Figure 3a shows the distribution of *BLISS_scores *as the shorter *G*_*score *profile was slid along the longer *G*_*score *profile. The peak of the *BLISS*_*score *indicates the maximum match. In this case, the abrupt surge in the *BLISS*_*score *is due to the match between the embedded hypothetical module in the short sequence and the conserved module in the long sequence. When this methodology was tested on all of the 10,000 simulated sequence pairs, about 80% of the highest peaks for each pair contained the correct match between the embedded hypothetical module and the conserved module.

### Distribution and statistical evaluation of the BLISS_score

To be able to evaluate the match at the binding site profile level, we analyzed the distribution of *BLISS_scores *using the simulated sequence pairs. For each pair of sequences, *BLISS_scores *were calculated at each position as the short profile slid along the longer profile. The peak matches (corresponding to the peaks in the score profile) between each pair of sequences were evaluated to see whether it aligned the embedded modules. If the match did align the modules, it was designated a "true" match. All other *BLISS_scores *were considered as background.

Figure 3b shows the distribution of the background and the "true" match *BLISS_scores *for the 10,000 simulated pairs of sequences. This distribution varies slightly depending upon the cut_off threshold set for *M*_*score *(Figure 3b&c). This is not surprising, since a lower cut_off threshold will lead to more identified potential binding sites and thus a slightly higher background score.

The distributions allow us to evaluate any particular *BLISS*_*score*. It is informative in helping set a threshold for reporting significant matches at the binding site level. Given a *BLISS_score x*, the distributions allow us to decide whether that *BLISS*_*score *corresponds to a true alignment of modules or whether it corresponds to the module aligning with a random DNA segment. Let *C1 *denote the event where the modules embedded in the short and the long sequences are aligned, and *C2 *denote the event where either module is aligned with a random DNA segment. Based on Bayes formula, the posterior probabilities can be calculated as follows:

p(C1|x)=p(x|C1)p(C1)p(x|C1)p(C1)+p(x|C2)p(C2)p(C2|x)=p(x|C2)p(C2)p(x|C1)p(C1)+p(x|C2)p(C2)
 MathType@MTEF@5@5@+=feaafiart1ev1aaatCvAUfKttLearuWrP9MDH5MBPbIqV92AaeXatLxBI9gBaebbnrfifHhDYfgasaacH8akY=wiFfYdH8Gipec8Eeeu0xXdbba9frFj0=OqFfea0dXdd9vqai=hGuQ8kuc9pgc9s8qqaq=dirpe0xb9q8qiLsFr0=vr0=vr0dc8meaabaqaciaacaGaaeqabaqabeGadaaakeaafaqabeGabaaabaGaemiCaaNaeiikaGIaem4qamKaeGymaeJaeiiFaWNaemiEaGNaeiykaKIaeyypa0ZaaSaaaeaacqWGWbaCcqGGOaakcqWG4baEcqGG8baFcqWGdbWqcqaIXaqmcqGGPaqkcqWGWbaCcqGGOaakcqWGdbWqcqaIXaqmcqGGPaqkaeaacqWGWbaCcqGGOaakcqWG4baEcqGG8baFcqWGdbWqcqaIXaqmcqGGPaqkcqWGWbaCcqGGOaakcqWGdbWqcqaIXaqmcqGGPaqkcqGHRaWkcqWGWbaCcqGGOaakcqWG4baEcqGG8baFcqWGdbWqcqaIYaGmcqGGPaqkcqWGWbaCcqGGOaakcqWGdbWqcqaIYaGmcqGGPaqkaaaabaGaemiCaaNaeiikaGIaem4qamKaeGOmaiJaeiiFaWNaemiEaGNaeiykaKIaeyypa0ZaaSaaaeaacqWGWbaCcqGGOaakcqWG4baEcqGG8baFcqWGdbWqcqaIYaGmcqGGPaqkcqWGWbaCcqGGOaakcqWGdbWqcqaIYaGmcqGGPaqkaeaacqWGWbaCcqGGOaakcqWG4baEcqGG8baFcqWGdbWqcqaIXaqmcqGGPaqkcqWGWbaCcqGGOaakcqWGdbWqcqaIXaqmcqGGPaqkcqGHRaWkcqWGWbaCcqGGOaakcqWG4baEcqGG8baFcqWGdbWqcqaIYaGmcqGGPaqkcqWGWbaCcqGGOaakcqWGdbWqcqaIYaGmcqGGPaqkaaaaaaaa@8FEB@

Where *p(C1|x) *is the conditional probability of *C1 *given a *BLISS_score x *and *p(C2|x) *is the conditional probability of *C2 *given a *BLISS_score x*; *p(C1) *is the prior probability of *C1 *and *p(C2) *is the prior probability of *C2*; *p(x|C1) *is the conditional probability of observing *x *given *C1 *and *p(x|C2) *is the conditional probability of observing *x *given *C2*. *p(x|C1) *and *p(x|C2) *can be read off directly from the distributions generated.

It is difficult to find the means to calculate the prior probabilities *p(C1) *and *p(C2)*. In this study, we assumed *p(C1) *= *p(C2)*, although we suspect that *p(C1) *might be smaller than *p(C2)*. This assumption allowed us to calculate the posterior probabilities to evaluate a *BLISS_score x*. In practice, we decided that *x *was a significant matching score if *p(C2|x) *was less than a threshold of, e.g. 0.01 or 0.001.

### Identifying a conserved regulatory module in distantly related species

To test the efficacy of the BLISS methodology in real sequences, we undertook the task of identifying the Even-skipped (eve) stripe 2 enhancer (S2E) in distantly related *Drosophila *species. Even-skipped, an important development regulatory gene in *Drosophila melanogaster (D.mela)*, is specifically expressed in seven transverse stripes in the embryo during the blastoderm stage. The stripe 2 enhancer is the best studied and includes TFBSs for five TFs, bicoid (Bcd), hunchback (Hb), giant (gt), Kruppel (Kr), and sloppy-paired (slp) [18-20]. Unfortunately, TRANSFAC 9.1 has matrices for only three of the five TFs, i.e., Hb, Kr, and Bcd. Our search was therefore limited in the sense that some of the participating TFBSs could not be predicted and used for the match comparison.

Previous experimental investigations have shown that S2E is evolutionarily conserved among *D. yakuba (D.yaku), D. erecta*, and *D. pseudoobscura (D.pseu) *[2,12,21]. All of these species are in the same subgenus (*Sophophora*) as *D.mela, with D.pseu *having the most distant relationship with *D.mela *(separated at about 40 million years ago). BLISS did identify the eve S2E modules among these four species. In particular, a significant peak was reported by BLISS when we searched the S2E module extracted from *D. pseu *against the 14 kb *D. mela *genomic sequence flanking the eve coding region.

**Figure 4:**
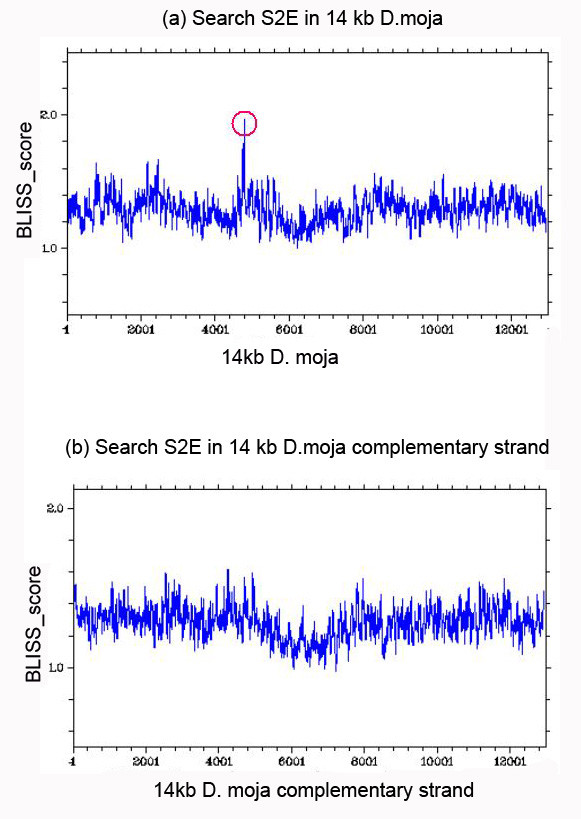
**Identifying the *eve *S2E module in distantly related species**. a.) Using the *D.pseu *S2E module (1027 bp), a peak (red circle) in *BLISS*_*score *was identified in a 14 kb *D.moja *genomic sequence surrounding the *eve *coding region. b.) No significant match was identified in the reverse strand (bottom panel) or an unrelated sequence (data not shown).

In contrast, no detailed information has been published about potential S2E in more distantly related species, such as *D. mojavensis (D.moja) or D. virilis (D.viri)*, both from a separate subgenus (*Drosophila*) separated from *D.mela *at about 60 million years ago [22]. To identify S2E in these two distantly related species, we extracted the 14 kb genomic sequence flanking the eve coding region from *D.moja *and *D.viri *genomic sequences. Blast analysis using the *D.mela *or *D.pseu *sequence harbouring the eve S2E module did not identify any significant match longer than 41 bp (using bl2seq with default gap penalty values). Using the BLISS methodology however, a significant peak in the *BLISS*_*score *was observed (Figure 4a, *p(C2|x) *< 0.05). Verification of this match indicated that it contained the TFBSs composing the S2E module. Similar results were obtained when corresponding sequence pairs involving 1.) D.mela and D.moja, and 2.) D.mela and D.viri, were analyzed. In contrast, no significant match was identified in the reverse-complemented sequence (Figure 4b) or in other 14 kb sequences unrelated to eve, indicating the specificity of the search.

**Figure 5:**
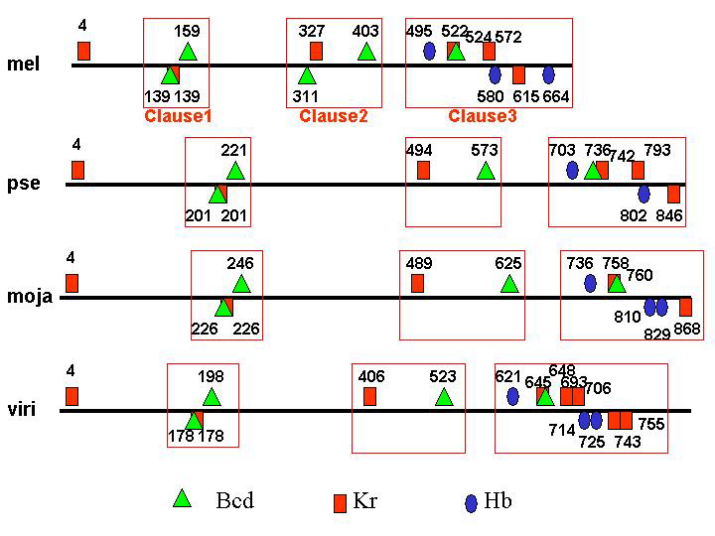
**Inter-TFBSs distances are very well conserved within each clause of the S2E module**. Comparison of the evolution of S2E modules across distantly related species revealed that while the sequence length of the module has changed significantly, the distance among TFBSs in Clauses 1 and 3 have remained stable. The numbers near the TFBS indicate the positions relative to the first Kr site in this module.

A detailed inspection of the make up of the S2E modules in distantly related species showed that S2E can be viewed as a complex module made of element modules. To make an analogy, S2E is a complex sentence that has several "clauses" (Figure 5). The evolution of the whole module indicates that the distances between some TFBSs have changed dramatically. However, the distances among the TFBSs within corresponding "clauses" have remained relatively stable. For example, in Clause 1 the distances among participating TFBSs have remained constant over the long evolutionary period. Specifically, the distance between the first bcd (overlapping with the first kr) and the second bcd is invariably 20 in all of the four species. In addition, the distances among TFBSs in Clause 3 have also remained relatively stable, i.e., within the variation we have factored into our simulation.

**Figure 6:**
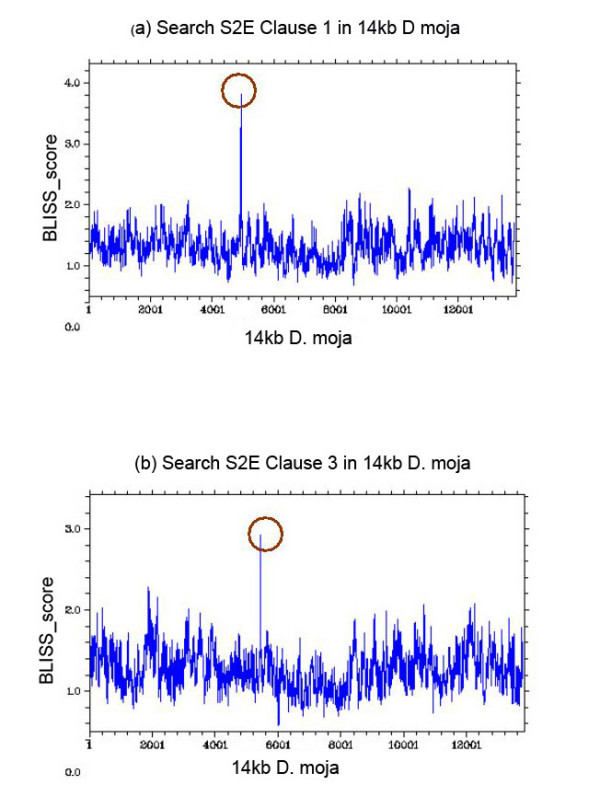
**Searching for conserved individual clauses/element modules using BLISS**. Profiles covering clauses 1 (a) or 3 (b) of S2E were used to search against a 14 kb D.moja genomic region. The *BLISS*_*score *peaks are significant (P(C2|x) = 0.0 for a, = 0.0003 for b).

Since our methodology is really based on the assumption of limited distance variations between TFBSs, it should be much more sensitive at identifying individual "Clauses" or simple modules. When the corresponding TFBS profile covering Clause 1 or Clause 3 were used to search against the genome sequence from *D.moja*, very significant peaks in *BLISS*_*score *were observed (Figure 6a&b, *p(C2|x) *< 0.001 for both). The peaks corresponded to the match of Clause 1 and Clause 3 on the *D.moja *sequence, respectively. BLAST analysis using the sequences covering Clause 1 or Clause 3 searched against the *D.moja *genomic sequence failed to identify significant matches that spanned the whole module. rVista 2.0 did predict Clause 1 because it succeeded in detecting the DNA similarity between the sequence covering Clause 1 and the *D.moja *sequence. However, rVista 2.0 failed to identify Clause 3 since no similarity was detected between the sequence covering Clause 3 and the *D.moja *genomic sequence.

### Implementation of BLISS as a web-based service

**Figure 7:**
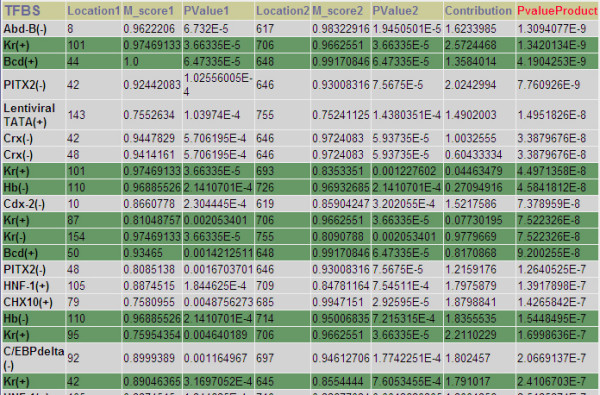
**BLISS output of the contributing TFBSs**. Our S2E search was used as the example. The list of TFBSs can be output either based on sequence position, product of p value (Figure 7), or contribution to *BLISS*_*score*. The TFBSs that belongs to S2E were highlighted in green.

The BLISS methodology has been implemented as a web-based tool for the research community. The web application embodies the Gaussian Smoothing Method for identifying *cis*-regulatory modules at the binding site level, and outputs all potential TFBSs in the predicted module. The module finding process consists of several steps:

To begin, the user inputs two DNA sequences. For example, a short sequence from a model organism that harbours a regulatory module, and a longer sequence surrounding the ortholog of a different species. An *M*_*score *threshold of 0.75 or 0.8 is then chosen by the user for the generation of the TFBS profiles for both sequences. Next, a plot of *BLISS_scores *comparing successive alignments of the short profile against the long profile is returned to the user. On the very same page, the distributions described earlier (Figure 3b&c) are displayed so that the user may choose a *BLISS*_*score *threshold. Once the *BLISS*_*score *threshold is chosen, BLISS outputs all of the matches with a *BLISS*_*score *higher than that threshold (limited up to 5). For each match, a table of contributing TFBSs are listed based on the product of the p-values of the matching TFBSs on both sequences (Figure 7). Alternatively, it can be listed based on their numeric contribution to the *BLISS*_*score*, or by the location of the TFBSs.

Currently, the limits for the short and long input sequences are set at 1200 bps and 15 k bps, respectively.

## Discussion

In this study, we have presented a first step towards identifying regulatory modules via comparisons at the binding site level. The advantage of such an approach is that it allows the detection of conserved regulatory modules in highly divergent sequences, as we have demonstrated both with simulated sequences as well as with real world examples. This method is thus complementary to many existing methods that are based on sequence similarity comparisons [23] or use sequence similarity for pre-analysis selection [4,5,7,24]. It should also be complementary to applications such as MEME and CompareProspector, which are widely used for the identification of conserved sequence motifs (binding sites) in the regulatory region of co-expressed genes [25,26].

There are limitations to our approach. Some of the major limitations, such as the coverage and quality of the TFBS matrices, are expected to improve rapidly in the near future as new high throughput techniques are applied to identify binding sites in genome scale. Our current algorithm is developed based on the assumption that the inter-TFBS distance variation is within a +/-4 base pair range. This allows the identification of modules/clauses with relatively small inter-TFBS distance variation, such as the individual clauses in the S2E module. It will likely miss modules/clauses that have much larger distance variations between TFBSs. In the case of S2E, the identification of the module was based on the fact that the third clause had low inter-TFBS distance variations, which was sufficient to generate a significant *BLISS*_*score *(figure 4a). As indicated by Ludwig et al, if S2E as a whole were to be considered, many inter-TFBS distances have changed dramatically during evolution [12]. However, a closer look at the distribution of TFBSs in S2E in the two distantly related species also indicated that the S2E module may be sub-divided into clauses (Figure 5). While the inter-clause distances have varied dramatically, the inter-TFBS distances within each clause have remained largely stable (Figure 5). This is very possibly a reflection of the spacing restriction on important transcription factor interactions.

In addition to the S2E module, we also tested our methodology on other regulatory modules such as the DME (Distal Muscle Enhancer) module in front of the paramyosin gene [27]. Using a 200 bp sequence harbouring the DME in *D.mela*, we were able to detect the corresponding module in *D.viri *(data not shown). Currently, the number of well characterized, evolutionarily conserved modules is limited. The goal of BLISS is to facilitate the discovery of multiple TFBSs modules by identifying the conserved pattern of the TFBSs. We also applied BLISS to a regulatory region that is responsible for mediating UV induced expression of *hac-1 *[28]. There is no existing information on the composition of the UV-responsive module in this region, which has very low sequence level conservation between the corresponding segments in *D.mela *and *D.pseu*. Yet genetic experiments have indicated that the responsiveness is highly conserved. The potential module identified by BLISS is currently being tested experimentally.

BLISS, with some adaptation, can potentially be used to identify the conserved regulatory modules in co-expressed genes. Another advantage of BLISS is that the methodology can also be applied to identify patterns that involve not only TFBSs, but also other sequence features such as complex response elements [29], insulator sequences, CpG islands, etc. Functionally, these sequence features (their related modifications and binding partners) interact with transcription factors. However, these features, such as CpG islands, cannot be detected by simple sequence similarity based searches.

## Conclusion

In this study, we addressed the feasibility of identifying conserved regulatory modules at the binding site level. Our results indicate that it is feasible to identify conserved regulatory modules in simulated random sequences harbouring a regulatory module made of 4–8 distinct binding sites. Using real sequences, we demonstrated that our approach outperforms regular sequence level comparisons when the orthologous DNA sequences are highly diverged. In addition, the BLISS program outputs directly the candidate binding sites that are shared between the two regulatory sequences, which can greatly facilitate the evaluation of the candidate module as well as the design of the experimental verification strategy by biomedical scientists. Future development of the project will include identifying better algorithms for complex modules and modules with higher inter-TFBS distance variations.

## Methods

### Generating simulated sequences

10000 simulated sequence pairs were generated for developing the methodology. Each set included a short DNA sequence (100–500 bps) harboring a hypothetical TFBS module and a long DNA sequence (5–6 kb) harboring a conserved TFBS module. First, the hypothetical TFBS module was generated in the following manner: 4–8 binding sites were randomly chosen from TRANSFAC 9.1 [15] database and then random DNA subsequences were inserted between them. Qiu et al. [16] analyzed all the entries of composite elements in the TransCompel database (version 3.0) and they found that about 87% of the composite elements are within 50 bp distance and about 65% are within 20 bp distance of one another. We therefore chose lengths of DNA subsequences inserted between binding sites based on this result. Next, we created the conserved TFBS module, which included binding sites for the same sets of transcription factors in the same order as in the shorter sequence. However, the binding site sequences had to be different and they were randomly chosen from TRANSFAC 9.1. Furthermore, compared to the hypothetical module, distances between binding sites were set to vary slightly and we allowed each binding site to shift up to 4 bps either to the right or to the left. Finally, we inserted the conserved module into a 5000 bp randomly generated DNA sequence to generate the longer sequence.

### Binding site identification

Potential TFBSs both in the short DNA sequence (including the hypothetical module) and the long DNA sequence (including the conserved module) were searched based on frequency matrices collected by TRANSFAC 9.1. Because TFBSs may be detectable on either the forward or the backward strand, we searched both strands of sequences. The *M*_*score *profile for each sequence is a M*L matrix, where M is twice of the number of matrices applied and L is the length of the sequence. The top half of the *M*_*score *matrix is the score profile for the forward strand and the bottom half is that for the complementary strand. The *M*_*score *of the *i*th TFBS at position *j *of the sequence was calculated by first aligning the frequency matrix for the *i*th TFBS with the sequence at position *j *and then computing:

f.1. M_score:

M_score[i,j]=Score[i,j]−ScoreMin[i,j]ScoreMax[i,j]−ScoreMin[i,j]Score[i,j]=∑k=0K−1I(k)fk,nj+kScoreMin[i]=∑k=0K−1I(k)fkmin⁡ScoreMax[i]=∑k=0K−1I(k)fkmax⁡I(k)=∑N∈{A,T,C,G}fk,Nln⁡(4fk,N)
 MathType@MTEF@5@5@+=feaafiart1ev1aaatCvAUfKttLearuWrP9MDH5MBPbIqV92AaeXatLxBI9gBaebbnrfifHhDYfgasaacH8akY=wiFfYdH8Gipec8Eeeu0xXdbba9frFj0=OqFfea0dXdd9vqai=hGuQ8kuc9pgc9s8qqaq=dirpe0xb9q8qiLsFr0=vr0=vr0dc8meaabaqaciaacaGaaeqabaqabeGadaaakeaafaqabeqbbaaaaeaacqWGnbqtcqGGFbWxcqWGZbWCcqWGJbWycqWGVbWBcqWGYbGCcqWGLbqzcqGGBbWwcqWGPbqAcqGGSaalcqWGQbGAcqGGDbqxcqGH9aqpdaWcaaqaaiabdofatjabdogaJjabd+gaVjabdkhaYjabdwgaLjabcUfaBjabdMgaPjabcYcaSiabdQgaQjabc2faDjabgkHiTiabdofatjabdogaJjabd+gaVjabdkhaYjabdwgaLnaaBaaaleaacqWGnbqtcqWGPbqAcqWGUbGBaeqaaOGaei4waSLaemyAaKMaeiilaWIaemOAaOMaeiyxa0fabaGaem4uamLaem4yamMaem4Ba8MaemOCaiNaemyzau2aaSbaaSqaaiabd2eanjabdggaHjabdIha4bqabaGccqGGBbWwcqWGPbqAcqGGSaalcqWGQbGAcqGGDbqxcqGHsislcqWGtbWucqWGJbWycqWGVbWBcqWGYbGCcqWGLbqzdaWgaaWcbaGaemyta0KaemyAaKMaemOBa4gabeaakiabcUfaBjabdMgaPjabcYcaSiabdQgaQjabc2faDbaaaeaacqWGtbWucqWGJbWycqWGVbWBcqWGYbGCcqWGLbqzcqGGBbWwcqWGPbqAcqGGSaalcqWGQbGAcqGGDbqxcqGH9aqpdaaeWbqaaiabdMeajjabcIcaOiabdUgaRjabcMcaPiabdAgaMnaaBaaaleaacqWGRbWAcqGGSaalcqWGUbGBdaWgaaadbaGaemOAaOMaey4kaSIaem4AaSgabeaaaSqabaaabaGaem4AaSMaeyypa0JaeGimaadabaGaem4saSKaeyOeI0IaeGymaedaniabggHiLdaakeaacqWGtbWucqWGJbWycqWGVbWBcqWGYbGCcqWGLbqzdaWgaaWcbaGaemyta0KaemyAaKMaemOBa4gabeaakiabcUfaBjabdMgaPjabc2faDjabg2da9maaqahabaGaemysaKKaeiikaGIaem4AaSMaeiykaKIaemOzay2aa0baaSqaaiabdUgaRbqaaiGbc2gaTjabcMgaPjabc6gaUbaaaeaacqWGRbWAcqGH9aqpcqaIWaamaeaacqWGlbWscqGHsislcqaIXaqma0GaeyyeIuoaaOqaaiabdofatjabdogaJjabd+gaVjabdkhaYjabdwgaLnaaBaaaleaacqWGnbqtcqWGHbqycqWG4baEaeqaaOGaei4waSLaemyAaKMaeiyxa0Laeyypa0ZaaabCaeaacqWGjbqscqGGOaakcqWGRbWAcqGGPaqkcqWGMbGzdaqhaaWcbaGaem4AaSgabaGagiyBa0MaeiyyaeMaeiiEaGhaaaqaaiabdUgaRjabg2da9iabicdaWaqaaiabdUealjabgkHiTiabigdaXaqdcqGHris5aaGcbaGaemysaKKaeiikaGIaem4AaSMaeiykaKIaeyypa0ZaaabuaeaacqWGMbGzdaWgaaWcbaGaem4AaSMaeiilaWIaemOta4eabeaaaeaacqWGobGtcqGHiiIZcqGG7bWEcqWGbbqqcqGGSaalcqWGubavcqGGSaalcqWGdbWqcqGGSaalcqWGhbWrcqGG9bqFaeqaniabggHiLdGccyGGSbaBcqGGUbGBcqGGOaakcqaI0aancqWGMbGzdaWgaaWcbaGaem4AaSMaeiilaWIaemOta4eabeaakiabcMcaPaaaaaa@0BD7@

*K *is the length of the TFBS. *n*_*j*+*k *_∈ {A,T,C,G} is the nucleotide occurring in the sequence at position *j*+*k*. fk,nj+k
 MathType@MTEF@5@5@+=feaafiart1ev1aaatCvAUfKttLearuWrP9MDH5MBPbIqV92AaeXatLxBI9gBaebbnrfifHhDYfgasaacH8akY=wiFfYdH8Gipec8Eeeu0xXdbba9frFj0=OqFfea0dXdd9vqai=hGuQ8kuc9pgc9s8qqaq=dirpe0xb9q8qiLsFr0=vr0=vr0dc8meaabaqaciaacaGaaeqabaqabeGadaaakeaacqWGMbGzdaWgaaWcbaGaem4AaSMaeiilaWIaemOBa42aaSbaaWqaaiabdQgaQjabgUcaRiabdUgaRbqabaaaleqaaaaa@35A7@ is the frequency of nucleotide *n*_*j*+*k *_at position *k *in the frequency matrix (of the *i*th TF). fkmin⁡
 MathType@MTEF@5@5@+=feaafiart1ev1aaatCvAUfKttLearuWrP9MDH5MBPbIqV92AaeXatLxBI9gBaebbnrfifHhDYfgasaacH8akY=wiFfYdH8Gipec8Eeeu0xXdbba9frFj0=OqFfea0dXdd9vqai=hGuQ8kuc9pgc9s8qqaq=dirpe0xb9q8qiLsFr0=vr0=vr0dc8meaabaqaciaacaGaaeqabaqabeGadaaakeaacqWGMbGzdaqhaaWcbaGaem4AaSgabaGagiyBa0MaeiyAaKMaeiOBa4gaaaaa@33AF@ is the lowest frequency and fkmax⁡
 MathType@MTEF@5@5@+=feaafiart1ev1aaatCvAUfKttLearuWrP9MDH5MBPbIqV92AaeXatLxBI9gBaebbnrfifHhDYfgasaacH8akY=wiFfYdH8Gipec8Eeeu0xXdbba9frFj0=OqFfea0dXdd9vqai=hGuQ8kuc9pgc9s8qqaq=dirpe0xb9q8qiLsFr0=vr0=vr0dc8meaabaqaciaacaGaaeqabaqabeGadaaakeaacqWGMbGzdaqhaaWcbaGaem4AaSgabaGagiyBa0MaeiyyaeMaeiiEaGhaaaaa@33B3@ is highest frequency across all nucleotides at position *k *in the frequency matrix (of the *i*th TF). *I(k) *is the information vector for the frequency matrix, which reflects the degree of conservation at position *k *of the matrix. Finally, *M_score [i,j] *is the normalized *Score [i,j]*. Stormo [30-32] observed that logarithms of the base frequencies ought to be proportional to the binding energy and the information vector reflects this average binding energy between the transcription factor and the binding site.

A score cutoff at 0.75 was applied to the *M*_*score *profiles of both the short and the long sequence as follows:

*M*_*score *[*i*, *j*] = *M*_*score *[*i*, *j*] if *M*_*score *[*i*, *j*] ≥ 0.75

*M*_*score *[*i*, *j*] = 0 if *M*_*score *[*i*, *j*] < 0.75

### p value for M_score

To calculate the p value, a background model is required. Here, we chose the background model to be a random DNA sequence where each position is drawn independently. The ratio among A, T, C, and G is 30% : 30% : 20 % : 20%. For each frequency matrix, 300 million subsequences were sampled from the background model, and the *M*_*score *of each subsequence was calculated to build the *M*_*score *distribution. The p value of a *M*_*score *for each TFBS was estimated by calculating the fraction of samples that had scores equal to or higher than that *M*_*score*. Then, the *P_score *profiles were calculated as follows:

f.2 P_score:

*P*_*score*[*i*, *j*] = *C*_i _* *M*_*score*[*i*, *j*]

*C*_*i *_is the negative natural logarithm of the p value of *M*_*score *> = 0.75 for the *i*th TFBS.

### Gaussian smoothing

To account for the change in the distances between/among binding sites, a Gaussian smoothing was applied to the *P_score *profiles with a variance of 9. Formally, the *G*_*score *profiles were calculated as follows:

f.3 G_score:

G_score[i,j]=∑k=−77P_score[i,j+k]*1σ2πe−k2/2σ2∑i=−771σ2πe−i2/2σ2
 MathType@MTEF@5@5@+=feaafiart1ev1aaatCvAUfKttLearuWrP9MDH5MBPbIqV92AaeXatLxBI9gBaebbnrfifHhDYfgasaacH8akY=wiFfYdH8Gipec8Eeeu0xXdbba9frFj0=OqFfea0dXdd9vqai=hGuQ8kuc9pgc9s8qqaq=dirpe0xb9q8qiLsFr0=vr0=vr0dc8meaabaqaciaacaGaaeqabaqabeGadaaakeaacqWGhbWrcqGGFbWxcqWGZbWCcqWGJbWycqWGVbWBcqWGYbGCcqWGLbqzcqGGBbWwcqWGPbqAcqGGSaalcqWGQbGAcqGGDbqxcqGH9aqpdaaeWbqaaiabdcfaqjabc+faFjabdohaZjabdogaJjabd+gaVjabdkhaYjabdwgaLjabcUfaBjabdMgaPjabcYcaSiabdQgaQjabgUcaRiabdUgaRjabc2faDjabcQcaQmaalaaabaWaaSaaaeaacqaIXaqmaeaaiiGacqWFdpWCdaGcaaqaaiabikdaYiab=b8aWbWcbeaaaaGccqWGLbqzdaahaaWcbeqaaiabgkHiTiabdUgaRnaaCaaameqabaGaeGOmaidaaSGaei4la8IaeGOmaiJae83Wdm3aaWbaaWqabeaacqaIYaGmaaaaaaGcbaWaaabCaeaadaWcaaqaaiabigdaXaqaaiab=n8aZnaakaaabaGaeGOmaiJae8hWdahaleqaaaaakiabdwgaLnaaCaaaleqabaGaeyOeI0IaemyAaK2aaWbaaWqabeaacqaIYaGmaaWccqGGVaWlcqaIYaGmcqWFdpWCdaahaaadbeqaaiabikdaYaaaaaaaleaacqWGPbqAcqGH9aqpcqGHsislcqaI3aWnaeaacqaI3aWna0GaeyyeIuoaaaaaleaacqWGRbWAcqGH9aqpcqGHsislcqaI3aWnaeaacqaI3aWna0GaeyyeIuoaaaa@7D3F@

where σ = 3 and *k *ranges from -7 to 7. In effect, a *P_score *can spread 7 positions to both the right and the left due to the Gaussian smoothing. Smoothed *P_scores *beyond 7 positions were ignored due to their small values.

### Searching the conserved module in the long sequence

To identify a maximum match at the binding site profile level, the short *G*_*score *profile was slid along the long *G*_*score *profile. *BLISS*_*score *at position *n *is the matching score between the short profile and its corresponding region of equal length (length of the short sequence) in the long profile at position *n*:

f.4 BLISS_score:

BLISS_score[n]=(∑i=0M−1∑j=0L−1G_score[i,j]*G_score2[i,j+n])/LengthOfShortSequence
 MathType@MTEF@5@5@+=feaafiart1ev1aaatCvAUfKttLearuWrP9MDH5MBPbIqV92AaeXatLxBI9gBaebbnrfifHhDYfgasaacH8akY=wiFfYdH8Gipec8Eeeu0xXdbba9frFj0=OqFfea0dXdd9vqai=hGuQ8kuc9pgc9s8qqaq=dirpe0xb9q8qiLsFr0=vr0=vr0dc8meaabaqaciaacaGaaeqabaqabeGadaaakqaabeqaaiabdkeacjabdYeamjabdMeajjabdofatjabdofatjabc+faFjabdohaZjabdogaJjabd+gaVjabdkhaYjabdwgaLjabcUfaBjabd6gaUjabc2faDjabg2da9aqaaiabcIcaOmaaqahabaWaaabCaeaacqWGhbWrcqGGFbWxcqWGZbWCcqWGJbWycqWGVbWBcqWGYbGCcqWGLbqzcqGGBbWwcqWGPbqAcqGGSaalcqWGQbGAcqGGDbqxcqGGQaGkcqWGhbWrcqGGFbWxcqWGZbWCcqWGJbWycqWGVbWBcqWGYbGCcqWGLbqzcqaIYaGmcqGGBbWwcqWGPbqAcqGGSaalcqWGQbGAcqGHRaWkcqWGUbGBcqGGDbqxcqGGPaqkcqGGVaWlcqWGmbatcqWGLbqzcqWGUbGBcqWGNbWzcqWG0baDcqWGObaAcqWGpbWtcqWGMbGzcqWGtbWucqWGObaAcqWGVbWBcqWGYbGCcqWG0baDcqWGtbWucqWGLbqzcqWGXbqCcqWG1bqDcqWGLbqzcqWGUbGBcqWGJbWycqWGLbqzaSqaaiabdQgaQjabg2da9iabicdaWaqaaiabdYeamjabgkHiTiabigdaXaqdcqGHris5aaWcbaGaemyAaKMaeyypa0JaeGimaadabaGaemyta0KaeyOeI0IaeGymaedaniabggHiLdaaaaa@91F0@

where *G*_*score1 *is the *G*_*score *profile for the short sequence and *G*_*score2 *is the *G*_*score *profile for the long sequence; *L *is the length of the short sequence; *n *is the current location where the short sequence is aligned to the long sequence.

### Large scale search of the simulated sequences, statistical analysis

We used 10000 simulated sequence pairs generated by the above method to calculate two *BLISS*_*score *distributions. The first is the *BLISS*_*score *distribution when the hypothetical module in the short sequence is aligned with the conserved module in the long sequence. The second is the *BLISS*_*score *distribution when the module is aligned with a non-module segment of the longer sequence. For each pair of sequences, *BLISS_scores *were calculated at each position as the short profile slid along the longer profile. The peak matches (corresponding to the peaks in the score profile) between each pair of sequences were evaluated to see whether it aligned the embedded modules. If the match did include the alignment of the modules, it was designated a "true" match, and this *BLISS*_*score *was used to calculate the distribution for the modules matching. All of the other *BLISS_scores *were used to calculate the distribution for the module matching with the background sequence.

### Searching for the eve2 module in D. virilis and D. mojavanis sequences

The GenBank [33] accession numbers for the S2E sequences are AF042712*(D. pseudoobscura) *and AF042709*(D. melanogaster)*. We used BLISS to search these two enhancers in 13 kb *D. virilis *and 14 kb *D*. mojavanis sequences, in which S2E is hypothesized to be located, but the specific location is unknown.

Ludwig et al. indicated that distances between TFBSs in two clauses in S2E (region 134–275 and region 484–684 for *D. melanogaster*, region 196–376 and region 692–866 for *D. pseudoobscura*) were substantially conserved. We removed those regions and searched for the modules in 13 kb *D. virilis *and 14 kb *D*. mojavanis sequences using BLISS.

### Website construction

BLISS was implemented using HTML/JSP/JavaBean and is supported by an Apache Tomcat 5.5 server. It is publicly available at: . The *M*_*score *profiles of TFBSs were calculated based on the frequency matrix library collected by TRANSFAC 9.1. BLISS used DISLIN [34], a plotting library for displaying data, to draw the match score plot in run time.

## Authors' contributions

H. Meng is the principal undertaker of this project. A. Banerjee provided guidance to HM in development of the algorithms and worked on the manuscript. L. Zhou initiated the project and provided general guidance to HM. HM and LZ drafted the manuscript.
